# The experience of infectologists faced with death and dying among their patients over the course of the AIDS epidemic in the city of São Paulo: qualitative study

**DOI:** 10.1590/S1516-31802010000200006

**Published:** 2010-03-04

**Authors:** Emi Shimma, Maria Cezira Fantini Nogueira-Martins, Luiz Antonio Nogueira-Martins

**Affiliations:** I BSc, MSc. Student of Mental Health, Universidade Federal de São Paulo (Unifesp) and psychologist at the Sexually-Transmitted Diseases-AIDS (STD-AIDS) Reference and Training Center, São Paulo, Brazil.; II BSc, PhD. Psychologist and researcher at the Institute of Health, State Health Department of São Paulo, São Paulo, Brazil.; III MD. Associate professor of Psychiatry and Medical Psychology, Department of Psychiatry, Universidade Federal de São Paulo (Unifesp), São Paulo, Brazil.

**Keywords:** Acquired immunodeficiency syndrome, Death, Grief, Patients, Physicians, Síndrome da imunodeficiência adquirida, Morte, Pesar, Pacientes, Médicos

## Abstract

**CONTEXT AND OBJECTIVE:**

With the emergence of the acquired immunodeficiency syndrome (AIDS) in 1981, infectologists’ care practices went through great changes. The objective of this study was to describe and analyze the experiences of infectologists in dealing with death and dying among their patients, over the course of the AIDS epidemic in the city of São Paulo.

**DESIGN AND SETTING:**

A qualitative approach was used. Twenty infectologists from five hospitals that treat human immunodeficiency virus (HIV)/AIDS patients in the municipality of São Paulo were interviewed.

**METHODS:**

The sample was formed through the snowball process. The sample size was determined using the saturation criterion. To analyze the material obtained from the interviews, the procedure of thematic analysis was used. This consisted of finding the core meaning relating to the study objective, within the set of materials obtained.

**RESULTS:**

Analysis of the material obtained from the interviews led to three main themes: 1. The initial context of AIDS and its impact on infectologists; 2. Modifications to the infectologists’ attachments to patients after the introduction of highly active antiretroviral therapy (HAART); 3. Coping with death and dying.

**CONCLUSIONS:**

This study shows the importance of considering the distress, emotional overload and adaptive mechanisms relating to death and dying among patients, both in training and in professional practice.

## RESUMO

**CONTEXTO E OBJETIVO:**

Com o surgimento da síndrome da imunodeficiência adquirida (aids), em 1981, os infectologistas passaram por grandes modificações em sua prática assistencial. Este estudo tem por objetivo descrever e analisar as vivências de médicos infectologistas frente à morte e ao morrer de seus pacientes ao longo da trajetória da epidemia de aids na cidade de São Paulo.

**TIPO DE ESTUDO E LOCAL:**

Adotou-se a abordagem qualitativa. Foram entrevistados 20 médicos infectologistas pertencentes a cinco instituições hospitalares que atendem portadores de vírus da imunodeficiência humana (HIV)/aids no município de São Paulo.

**MÉTODOS:**

A composição da amostra foi realizada pelo processo de *snowball* e o tamanho da amostra determinado pelo critério de saturação. Para a análise do material obtido nas entrevistas, foi utilizado o procedimento da *análise temática,* que consiste em descobrir os núcleos de sentido existentes no conjunto do material obtido e que têm relação com o objetivo do estudo.

**RESULTADOS:**

Da análise do material obtido nas entrevistas resultaram três eixos temáticos: 1. O contexto inicial da aids e o impacto vivido pelos infectologistas; 2. Modificações na vinculação com os pacientes depois da introdução da terapia antirretroviral potente (HAART); 3. Lidando com a morte e o morrer.

**CONCLUSÕES:**

O estudo aponta para a importância de considerar, tanto na formação como no exercício profissional, o sofrimento, a sobrecarga emocional e os mecanismos adaptativos relacionados à morte e o morrer de pacientes.

## INTRODUCTION

Thirty-three million people were estimated to be living with human immunodeficiency virus/acquired immunodeficiency syndrome (HIV/AIDS) around the world in 2008. In that year, 2.5 million new cases of infection by HIV and 2.1 million deaths due to AIDS were registered.^1^

The first cases of AIDS in Brazil were diagnosed in the city of São Paulo, in the early 1980s. In the state of São Paulo, between 1980 and June 2008, 171,647 cases with 87,050 deaths were reported.^2^ In the whole of Brazil, over the same period, 506,499 AIDS cases were registered, with 205,409 deaths. Today, the epidemic is considered stable in this country.^3^ The state of São Paulo accounts for 40% of the cases reported in Brazil, and the city of São Paulo has 39% of the total number of cases in the state of São Paulo.

The transmission pattern has changed over the course of the epidemic. In the state of São Paulo, throughout the 1980s, the predominant exposure category was homosexual and bisexual. AIDS cases among drug users appeared in 1986 and remained high until 1991; from that year onwards, this rate started to drop. Starting in 1990, AIDS cases among heterosexuals gradually grew in absolute numbers. Since 1987, there has been a growing trend of AIDS cases among women.^2^

Since the introduction of highly active antiretroviral therapy (HAART) in Brazil in 1996, AIDS has been considered to be a chronic disease. The survival of AIDS patients of more than 12 years of age was 5.1 months over the period from 1982 to 1989, 16 months in 1995, and 58 months in 1996. In the southern and southeastern regions of Brazil, about 60% of the patients who were diagnosed with AIDS between 1998 and 1999 survived more than 108 months.^3^ In spite of the disparities between the country’s regions, universal access to HAART has contributed towards a fall in AIDS mortality, and is thought to have contributed towards a decline in incidence of the disease.^4^

Today, deaths due to AIDS result from late diagnosis and therapeutic failure. Compliance with antiretroviral treatment and early diagnosis of HIV-positive individuals are the current main challenges.^5^

The course followed by AIDS involves many social players (patients, families, physicians, healthcare workers in general and researchers) and also many stories of loss and mourning. Dealing with death is certainly one of the greatest challenges that humans face. Awareness of the finiteness of life and anguish when faced with the unknown have driven humankind to build religions and superior and immortal beings, as a clear projection of the human wish to find refuge and comfort from death.

Faced naturally until the early 20^th^ century, death has since become regarded as an enemy to be defeated and is generally associated with physical decrepitude. With the development of new technologies and powerful medications, medicine has become essentially centered on providing cures and fighting death at any cost. Physicians have gradually been delegated the role of "saviors": people who are capable of fighting against death and, maybe, defeating it, like the gods can. Physicians do not have this power. When faced with a terminal patient, physicians tend to feel uncomfortable and powerless, like any other human being. They are trained in schools that share the reference points of today’s society, i.e. that death is a sign of failure.^6^

In addition to the sociocultural aspects of the issue of death, there are also psychological aspects. The death of people to whom there was an emotional attachment is usually accompanied by a physical and psychological state characterized by a set of sensations, thoughts and feelings that are called mourning. This is a natural and universal emotional reaction to loss, which involves psychological, physiological and behavioral symptoms.^7^ It is also a stressful situation that is faced in attempting to adapt to a new reality.

An individual’s reaction to a loss due to death depends on a number of factors: the nature of the bond with the deceased, the type of death (expected or sudden), the person’s repertoire of coping techniques, previous experiences, capacity to tolerate strong emotions, the age of the deceased and this person’s role in the family system.^7^

Usually, a person going through a mourning process goes through four basic phases: numbness; searching and yearning; despair; and recovery. These phases are part of normal mourning.^8^ The duration and intensity of these phases, along with their order of manifestation, many vary according to each individual’s personality and predisposition: age, sex, self-esteem, life history, meaning of loss, bond between the deceased and mourner, previous losses that were poorly worked through, type of death, and social support network. Regarding the type of mourning, it can be: normal; chronic (indefinite extent of mourning); delayed (mourning is held back until it emerges as an overreaction after a later loss); or inhibited (absence of normal mourning symptoms).^7^

It can be said that the mourning process ends when the person feels adapted to the loss, i.e. feels able to take on new roles, make new emotional investments and give new meaning to life.^7-9^

## OBJECTIVE

This study has the objective of describing and analyzing the experiences of infectologists in dealing with death and dying among their patients, over the course of the AIDS epidemic in the city of São Paulo.

## POPULATION AND METHODS

This study used a qualitative approach,^10,11^ which made it possible to investigate phenomena relating to subjectivity and intersubjectivity, thereby going deeper into the meanings of actions, behavior and human relations.

The sample was formed through the snowball process, which provides a study sample based on referrals made by individuals who share the characteristics to be considered in the investigation or who know others with these traits.^10^ The sample size was determined by means of the criterion of saturation,^10^ i.e. the investigation ended when the interviews started to show content repetition. The project was approved by the research ethics committee of Universidade Federal de São Paulo (Unifesp).

The inclusion criteria for the study participants were that they needed to be infectologist experts and to have worked and still be working within the field of AIDS in public health services in the city of São Paulo.

The interviews were conducted by the first author. During the semi-structured interviews, both verbal and non-verbal expression was considered. To register verbal expression, a recorder was used; a logbook was used to write notes about non-verbal expression. The interviews were held in places chosen by the interviewees. The interviewees were asked to speak freely about situations of death among patients with AIDS and about how they had seen themselves when faced with such cases.

To analyze the material provided by the interviews, the procedure of thematic analysis was used. This consisted of finding core meanings relating to the study objective, from the material obtained.^11^

## RESULTS

Five infectologists with renowned AIDS experience were initially interviewed. At the end of the interviews, each professional was asked to indicate other infectologists, in order to allow the investigation to continue. Eighteen physicians were contacted for the next stage of the investigation, but three of them did not participate in the study because they claimed that they lacked the time.

The final sample was made up of 20 physicians (10 men and 10 women), who belonged to five hospitals that treat HIV/AIDS patients in the city of São Paulo. The average age was 51 years, and the average length of experience in the field of AIDS was 22 years.

Sixteen interviews were held at the physicians’ institutional workplaces, while three others preferred to be interviewed at their private clinics. One physician asked to be interviewed at home. The average duration of the interview was 60 minutes.

The infectologists showed themselves to be engaged in the theme and were quite open when dealing with their professional experiences relating to death and dying among their patients. Some interviewees expressed appreciation for the opportunity to talk and think about situations relating to their professional activity.

The number of interviews was considered sufficient when the interviews started to show content repetition.

The interviews were transcribed. The three authors read them and were jointly responsible for the analysis.

Analysis of the material obtained from the interviews led to three main themes:

1. The initial context of AIDS and its impact on infectologists;
2. Modifications to the infectologists’ attachments to patients after the introduction of HAART;
3. Coping with death and dying.

## DISCUSSION

The topic of death and dying expresses well the issue of duality in human behavior: although it is a subject that is usually avoided (as a taboo), it is also, when dealt with respectfully, a topic that gives rise to interest and desire to express feelings, ideas, beliefs and opinions.^10^ Thus, the analysis categories chosen represent possible angles within the rich and wide-ranging material provided by the study participants.

### 1. The initial context of AIDS and its impact on infectologists

The emergence of the epidemic in the early 1980s affected humankind profoundly. Among healthcare workers, a set of psychological reactions and many ethical issues permeated their care for patients. Physicians expressed the fears, conflicts, dilemmas, anxieties and limitations inherent to humankind, in different ways. They witnessed multiple deaths and were divided between providing care and help and the fear of becoming infected by something that was still unknown.^12,13^

Among the experiences and behavior observed at that time, the most prominent were: fear of contagion, avoidance, full identification with patients, distancing and discomfort relating to the affected individuals’ lifestyle. These behavioral patterns tended to occur to a greater degree among workers who were looking after patients with behavioral abnormalities, such as psychotic episodes and dementia. Thus, infectologists had to simultaneously deal with issues involving fears, prejudice, sexuality, drug abuse, madness and death, which they did not feel they had the tools to deal with.^14^

*"… We had to change our whole mindset… Dealing with a situation we didn’t know about, with a population segregated from society, that used drugs; homosexuals. It was a mess in our minds."*

Death was experienced as failure, thereby generating frustration and inducing depressive states.

*"I graduated in 1979. All of a sudden, the AIDS pandemic started… then everything changed… I was faced with a very difficult situation, because sick people died… I was depressed, down… and these patients died ‘in batches’, so to speak".*

*"…In the beginning, there were so many people dying that I was emotionally overwhelmed… Death was greater than the knowledge of physicians… AIDS was above medicine… I went through periods of great fragility… even a certain chronic depression…"*

In the early days of the epidemic, faced with the large number of deaths within a short period of time and the lack of specific drugs, infectologists had to deal with feelings of bewilderment, anguish and powerlessness.

*"… the first phase [of the epidemic], without treatment… was a difficult phase for us, infectologists… All of a sudden, we were dealing with a disease for which we already knew what the outcome would be and for which we were unprepared…"*

*"I went through all the early days of the epidemic… it was very sad… back then, we hospitalized five, six people… there was a line to die… with the whole Calvary that came along with dying from AIDS: dying paralyzed, dying blind, dying with diarrhea, wanting to die… dying demented, the family wanting the patient to die…"*

It is known that working through the emotional impact of those losses (which, it should be remembered, were intense and condensed) is very important for psychological health and to prevent disorders among workers. Physicians’ failure to acknowledge the psychological states resulting from the impact of patients’ deaths and low self-awareness and poor consciousness of their own emotions and feelings (especially psychological distress) might contribute towards development of psychosomatic symptoms, inadequate or inefficient adaptive mechanisms and burnout.^15-17^

It is also known that working through deep wounds and emotional pain demands time and consumes psychological energy. At that time, because of the speed of events, physicians did not have much time available to metabolize the painful impact of those losses and, in this manner, the mourning process was constantly fragmented.^12,13,18^ Combined with these factors, since death is a reason for embarrassment, physicians did not always allow themselves to lament the loss of patients. They usually experienced what is called "unauthorized mourning".^19^

*"… there were very sad times during the epidemic. There was not a single week without us losing patients… there were as many as four, five, six patients hospitalized at the same time, all very severely ill… we had to keep death certificate forms at home… they all died."*

Conflicts or ethical dilemmas (making clinical decisions involving ethical issues and values) arose overwhelmingly: the moral obligation to provide quality care required that physicians need to manage their fears and prejudices. The limits of professional capacity and the absence of treatment alternatives led to doubts about the investment in expensive and ineffective medications. The diagnosing of a terminal state brought up issues relating to continuing or interrupting treatment.

*"… I and other colleagues had a clear position that it was not fair to families to keep on after a certain point, but this led to distress [when I thought about whether] I had the right to do that [to sedate] or did not have the right to do that… there wasn’t a manual providing references in a clear way… The level of distress in making a decision such as this [to sedate] was huge, because you turn on that thing… and in five days, at most, the creature would die…"*

Despair led some HIV/AIDS patients to suicide, thereby generating feelings of impotence, frustration, incompetence, fragility, guilt and depression among the infectologists that were providing care for them.

*"…They took the lead [committing suicide]. It was unpredictable; [these situations] were more traumatic to me than the deaths during the natural course of the disease… the impact of unexpected news… and always questions: ‘could I have done anything else to avoid this?’…"*

### 2. Modifications to the infectologists’ attachments to patients after the introduction of HAART

Since 1996, thanks to the introduction of HAART, there has been a significant increase in the survival of HIV-positive patients. As a result of this increase in survival, the attachment between physicians and patients became longer and potentially deeper. Today, death, when it happens, takes place after a long period of interaction between professionals, patients and families, leading predominantly to feelings of sadness.

*"… Patients rarely die suddenly… Just as families have time to gradually prepare, I too have time to prepare myself. [With] some of them, I get really sad, they are very close…"*

Even though they realized that they had done their best, some professionals did not hide their frustration.

*"… every loss is a real failure for a physician. It means not having managed to accomplish the mission that we set out to do… If it were up to me, no one would die, if I could…"*

The closer bonds between physicians and patients favored the development of intense identification processes.

*"… we are more affected if their age is closer… I think that you project yourself a little… You compare yourself, you see that life is not eternal. You feel the fragility of human beings, which in fact, you also have in yourself…."*

In the case of children, a greater impact is noted.^20^ The death of children or young people who have been treated by infectologists, sometimes since birth, causes strong emotional impacts, because of the breakage of the emotional bond that was built up and because of the interruption of the natural cycle of life.

*"… today, losing a patient with AIDS is something terrible, because people no longer die of AIDS… today it’s equally dramatic, like the first death… I suffer a lot, I cry, I don’t want [the children] to die… if we manage to treat them and the virus is not of the worst type, I manage to make them survive until adulthood and I don’t know how long…"*

Some physicians openly admitted their difficulty in witnessing the dying process of young people with AIDS, because of the bond. This situation recalls the study by Malbergier and Stempliuk,^21^ which found that, out of 17 infectologists who were treating AIDS patients, only four felt that they were ready to cope with the issue of death.

*"… I couldn’t go to see her… and this bothered me because it felt like abandonment, you know. A girl that you saw many times and you couldn’t go to her room because she was dying and it was really unfair…. [I was] totally unprepared to deal with the pain"*

Infectologists who treat adults still have some difficulty in dealing with death, probably because it places them face to face with their own mortality and vulnerability, thereby arousing feelings of fear, irritation and frustration.^22^^-^^26^

*"… I don’t deal well with death, I don’t like the dead… confirming a death, for me, is very difficult… I don’t like it, but I have to go… it feels like death to me when I can’t reverse (the situation). I feel very bad, I don’t deal well with death… I don’t accept death"*

For several physicians, the process of dying was considered more difficult, impactful and touching than death itself. This period, called anticipatory grief, involves a whole range of emotional responses that may include adaptation to a new situation, separation anxiety, existential loneliness, sadness, disappointment, anger, resentment, guilt, exhaustion and desperation.^7^

*"I think that the most difficult thing is to follow up the process… as it evolves, you prepare yourself, somehow… you end up preparing not just yourself, but the family too."*

*"… Now [since HAART came in] it’s worse… you followed them up, saw them grow… the bond is huge… it’s very, very, very difficult… more than death, we are afraid of suffering… [What] hurts is to see people dying, more than death itself"*

For some respondents, the death of patients was still distressing, but in a more attenuated form and with more acceptance.

*"… You see [death] with acceptance… there isn’t that thing of insisting… you take them to the ICU, you get to a point when you talk to the family: ‘Look, there is nothing else to be done, no prospects…"*

*"… She was a patient I liked a lot; I saw that she struggled a lot, in spite of having problems with medications… there was nothing else I could do for her, none of the drugs available worked, then she got to my limit and I had to end up accepting this, because unfortunately, this is the way it is: HIV has no cure."*

The possibilities that attending physicians have for resorting to palliative care teams in organizations has been emphasized as an important resource today.

*"There’s the palliative care team… [this form of care] is relatively recent… it is extremely useful… As it is a multidisciplinary team, you can discuss everything that’s involved; you can share responsibilities. So, you can know whether it’s time to give up or not. [You can think together] how you will make the process lighter for the patient. [You can] make joint decisions to sedate the patient…"*

Some of the respondents showed frustration and tiredness after so many years on this path. Caring for HIV/AIDS patients today, in spite of the resources, may be painful. Despite the fulfilling nature of the care provided for patients with AIDS, it also creates burnout. Living with losses is intense and the prospects are discouraging, which leads some workers to give up fighting. Care may be even more difficult if the organizational structure does not offer support and acceptance for workers.^13^

*"…it is a complicated disease: to deal with patients, with all the paper work… it’s a disease you never cure…. your practice will have more and more sick people, you won’t solve things… in addition to not solving the problem definitively, things add up: every day you will have more and more sick people, except when someone dies… so, it’s frustrating, both professionally and in terms of workload".*

### 3. Coping with death and dying

According to Parkes^7^ and Redinbaugh et al.,^27^ the impact of patients’ deaths correlates with the following factors: how death occurred, the characteristics of the deceased, professionals’ identification with patients and their families, professional performance assessment in providing care for patients, the feeling of responsibility for death, quality of the bond with patients, duration of the bond, individual experience with death and personal history and vulnerabilities.

When faced with the impact of death and dying, humans usually use certain mechanisms, resources or strategies in order to work through and/or process these events. One of the most common working mechanisms is known as rationalization. This mechanism is characterized by achieving balances and reflections about the events that occurred, through ideational resources. In essence, these are the settings for the considerations and evaluations that the person makes regarding the circumstances that surrounded the events and their participation in them. Some accounts exemplified the use of this mechanism.

*"…I try to do all I know… It happened, I am sorry, and that’s it. That’s it, that’s it; let’s move on… We need to know to move on, or we’ll get crazy…"*

*"… everyone is sad when they lose a patient, but I think that we are left at least with the certainty that we did all that was possible; this relieves the loss. I tried everything… I’ve run out of possibilities…"*

*"… death is part of my profession and I have come to realize, the hard way and not consciously, that if every time a patient died I were to die a little, I wouldn’t be able to put up with it… I’ve learned to become harder, more rational, to see death in a different way…"*

Another resource that is frequently used is the creation of a kind of protective layer that, in some critical situations, is fundamental for preserving psychological integrity. However, it may develop into a problem if it turns into rigid armor that is impermeable to emotions and feelings and that becomes part of the personality and influences the way the person relates with life and other people. The use of this mechanism emerged in some accounts.

*"… I was a resident and worked in an ICU. I went through all these aspects of AIDS right through this time… we can say that I’ve developed armor, a shell… of self-protection… I rationalize… I think that life and death are part of being human… [Upon the death of a patient], I see another patient… I don’t think too much about that major loss I had… all this is part of my shell, of my shield…"*

*"… It is a defense you make. Unfortunately, we gradually get very cold. I don’t know whether unfortunately or not, but there are very few patients who affect us more deeply. We don’t lose many patients often… one dies every 3-6 months… Today, it’s kind of mechanical. You do everything up to a certain point; there is a moment when you are left with no drugs to use…"*

Some respondents reported having the need to compensate, somehow, for the suffering experienced with patients.

*"… there was a time when I used to go out shopping, to spend money. I had to fill myself with presents to put up with it. I thought I deserved it… it was clear, I was conscious of it: ‘I’ll buy that thing I’ve been wanting for a long time, because I deserve it. But today, I don’t do that any longer, at least not so intensely."*

Activities relating to technical-scientific improvement were also mentioned as a way of coping with losses and being ready to deal with future situations. It is known that the fear of making mistakes is simultaneously one of the most stressing factors associated with medical practice and one of the most powerful drivers for professional improvement.

*"… [so], I try to keep abreast, up-to-date, to study, to do the best for my patients, so they can believe me."*

*"… First I get sad, then I get grumpy, and nothing is fun to me… [I study], work on the case… if I find that I am guilty of anything, it gets even worse… [then] I spend a few days thinking, wondering…"*

Religious rituals and beliefs also contributed towards coping with situations of death among patients.

*"Rites are really helpful for me: I sing and dance to the dead. When a patient is close, if I can, I go to the funeral, the seventh-day mass… Rituals are really helpful… they have existed for so many thousands of years…"*

*"… the fact that you believe in reincarnation helps a lot… to believe that there is a bond, a pathway, that you are continuously improving… I think that it helps to accept many things."*

The acceptance of death as an inexorable fact was also mentioned in the interviews.

*"… I see death as part of life; I don’t see it as suffering. The important thing is to be aware that you did all that was necessary to avoid distress. I accept it. Previously, I was not aware of what dying was. In the beginning I suffered. It was horrible, but then I accepted it.*

Peer support for the work done in institutions, and individual psychotherapy support were considered important resources for coping with difficult situations.

*"… here [hospital] is a place that offers some peace, because there are other people to share things with… if you get a more complicated case… you can discuss it here… people give opinions; you record them. So, it is very different from being alone in your office."*

*"… maybe the great difference was being able to deal with my powerlessness, that is, my clear delusions of omnipotence, which every physician has… I could see them… it’s been a long way… I am absolutely convinced that it would have been impossible without the help of psychoanalysis, which I had all the time."*

There are, therefore, many possibilities and ways of coping with deaths among patients. Like in the study by Moores et al.,^28^ the physicians interviewed adopted different types of mechanisms for coping with losses. The literature discusses measures for facilitating and contributing towards physicians’ abilities to process and cope with the emotions and feelings associated with death and dying among their patients.^16,17,24-28^ There are data indicating that workers who do not feel prepared to cope with these situations tend to develop a broad range of reactive feelings, such as: professional loneliness, loss of the sense of mission, cynicism, desperation, frustration and an increased risk of burnout and depression.^16,17^

The acknowledgement that medicine is a potentially stressful occupation, has led to increases in prophylactic and/or care measures for physicians and other healthcare workers, over past decades. Interest in and concern with these issues has led to significant growth in studies on the vicissitudes of medical education and professional practice,^14-18^ with development of health promotion actions, early detection of emotional and occupational disorders and creation of care programs specific for students, residents and physicians.^29-31^ It should be highlighted that, within the field of organization of health services, the introduction of palliative care teams is currently considered to be a priority measure.^17^

## FINAL CONSIDERATIONS

Humankind, infectology and infectologists have gone through deep changes with the emergence of the HIV/AIDS epidemic. Before the HAART era, the large number of deaths led to feelings of impotence, distress and frustration. The infectologists interviewed (who, it should be emphasized, belong to the group of physicians who continued to work in this field) have managed, in different ways, to cope with the impact of those losses. They reported the need to share their experiences and to take care of their own mental health.

After the introduction of HAART, the scenario changed, emotionally speaking, to a context in which there are fewer losses. The bonds established with AIDS patients last longer and may, for example, trigger more intense identification with patients and with their families, thus leading to a psychological configuration that needs to be managed by the professional and by the organization, so as to enable good medical follow-up.

## CONCLUSIONS

In short, this study shows the importance of considering the distress, emotional burden and adaptive mechanisms relating to death and dying among patients, both in training and in professional practice. By encouraging debate and research, healthcare organizations can contribute towards furthering knowledge in the field of occupational stress; outlining preventive strategies against occupational disorders; expanding psychological care programs for students, residents and physicians; and, consequently, improving the quality of the care that they provide for patients.
